# The association between vitamin D and uterine fibroids: A mendelian randomization study

**DOI:** 10.3389/fgene.2022.1013192

**Published:** 2022-09-21

**Authors:** Weijie Guo, Mengyuan Dai, Zhuoling Zhong, San Zhu, Guidong Gong, Mei Chen, Junling Guo, Yaoyao Zhang

**Affiliations:** ^1^ Department of Obstetrics and Gynecology of West China Second University Hospital, BMI Center for Biomass Materials and Nanointerfaces, College of Biomass Science and Engineering, Sichuan University, Chengdu, Sichuan, China; ^2^ Key Laboratory of Birth Defects and Related of Women and Children of Ministry of Education, West China Second University Hospital, Sichuan University, Chengdu, China; ^3^ State Key Laboratory of Polymer Materials Engineering, Sichuan University, Chengdu, Sichuan, China

**Keywords:** vitamin D, uterine fibroids, SNP, mendelian randomization, GWAS

## Abstract

Uterine fibroids (UFs), the most common benign gynecological tumor, can bring severe negative impacts on a woman’s life quality. Vitamin D, is thought to play an important role in regulating cell proliferation and differentiation. In recent years, several studies suggested that higher level of vitamin D has a negative effect on the occurrence of UFs, but the results of studies on the relationship between them are conflicting and further evidence needs to be studied. Here in, we used a two-sample Mendelian Randomization (2SMR) study to explore the causal relationship between genetically predicted vitamin D levels and the risk of UFs. The exposure data comes from a genome-wide association study (GWAS) summary dataset consisting of 441,291 individuals, which includes datasets from United Kingdom Biobank, FinnGen Biobank and the corresponding consortia. Single-nucleotide polymorphisms (SNPs) associated with vitamin D at a significant level of *p* < 5 × 10^−8^ and low linkage disequilibrium (LD) level (r^2^ < 0.01) were selected. The outcome data comes from a GWAS dataset of IEU analysis of United Kingdom Biobank phenotypes consisting of 7,122 UFs cases and 455,811 controls. Our inverse-variance weight (IVW) analysis results support the causal association of genetically predicted vitamin D with the risk of UFs (OR = 0.995,95% CI = 0.990-0.999, *p* = 0.024). In addition, heterogeneity and pleiotropy were not observed in statistical models. In summary, our results indicate that elevated serum vitamin D levels are in strong relationship with reduction of the risk of UFs, which indicates that the clinical treatment of UFs may have a new and excellent option.

## Introduction

Uterine fibroids (UFs), also known as uterine leiomyomas, are benign tumors that negatively affect the function of the uterus in women of childbearing age. The most common symptom of UFs is heavy menstrual bleeding and the resulting anemia and pain (Kempson and Hendrickson, 2000). Other symptoms include pelvic pressure and pain, urinary incontinence and retention, and bowel dysfunction also place a significant burden on patients. UFs may also cause several reproductive problems, such as impaired fertility, pregnancy complications, miscarriage and adverse pregnancy outcomes (Wallach et al., 1981; Zimmermann et al., 2012; Khan et al., 2014; Vercellini and Frattaruolo, 2017). A study pointed out that UFs account for 1/3 to 1/2 of the reasons for hysterectomy and are the most common reason of hysterectomy in the United States (Stewart et al., 2016). Risk factors for UFs have received increasing attention in order to better prevent the occurrence of UFs. Many studies indicated that vitamin D deficiency increases the risk of UFs (Baird et al., 2013; Sabry et al., 2013; Ciebiera et al., 2016). There is a clinical study indicating that vitamin D supplementation has a significant therapeutic effect in patients with small UFs (Ciavattini et al., 2016). However, another clinical trial suggested that vitamin D levels have no significant effect on the occurrence of UFs (Arjeh et al., 2020). Overall, the relationship between vitamin D levels and the risk of UFs is still ambiguous, and further studies are needed to be carried out.

Vitamin D, a fat-soluble vitamin, is a general term for a group of structurally similar sterol derivatives. The target organs of vitamin D are widely distributed, and different type of vitamin D binds to their specific receptors and then play different roles (Lips, 2006). Vitamin D3 and vitamin D2 are the most important members of vitamin D (Ciebiera et al., 2018), and vitamin D3 is the main form of vitamin D in the human body. The level of vitamin D3 in serum can represent the total content of vitamin D in the body and the strength of the effect of vitamin D on the human body (Lips, 2006). Therefore, in the work, we focus on the level of vitamin D3 in serum. vitamin D3 has an anti-proliferative effect and can accelerate the release of tumor necrosis factors from macrophages, which has a broad killing effect on tumor cells (Van Den Bemd et al., 2000; Lips, 2006). Several recent studies pointed out that vitamin D deficiency is an important risk factor for UFs (Baird et al., 2013; Sabry et al., 2013; Ciebiera et al., 2016), and animal experiments have also shown that high doses of vitamin D can decrease the size of UFs (Al-Hendy and Badr, 2014; Ali et al., 2020), but the relationship between vitamin D and the pathogenesis of UFs or a positive and significant treatment effect remains unclear.

Mendelian randomization (MR) studies are conducted based on Mendel’s laws of inheritance and the use of instrumental variables (IVs). Mendelian laws of inheritance state that genes are randomly assigned and freely selected in the process of inheritance, instrumental variables are related to the risk factors we are interested in but not related to other confounding factors, and its effect on the outcome can only be determined by the exposure factor (Burgess and Thomson, 2015). Therefore, in the two-sample MR study, we use genetic variables to analyze the causal relationship between exposure factors and outcomes, typically single-nucleotide polymorphisms (SNPs). Due to the genes assigned during pregnancy, the direction of the causal relationship can also be determined. The association between serum vitamin D3 levels and the occurrence of UFs has not previously been studied using MR. In this study, we focused on exploring the causal relationship between serum vitamin D3 levels and the occurrence of UFs.

## Materials and methods

### Study design

**FIGURE 1:**
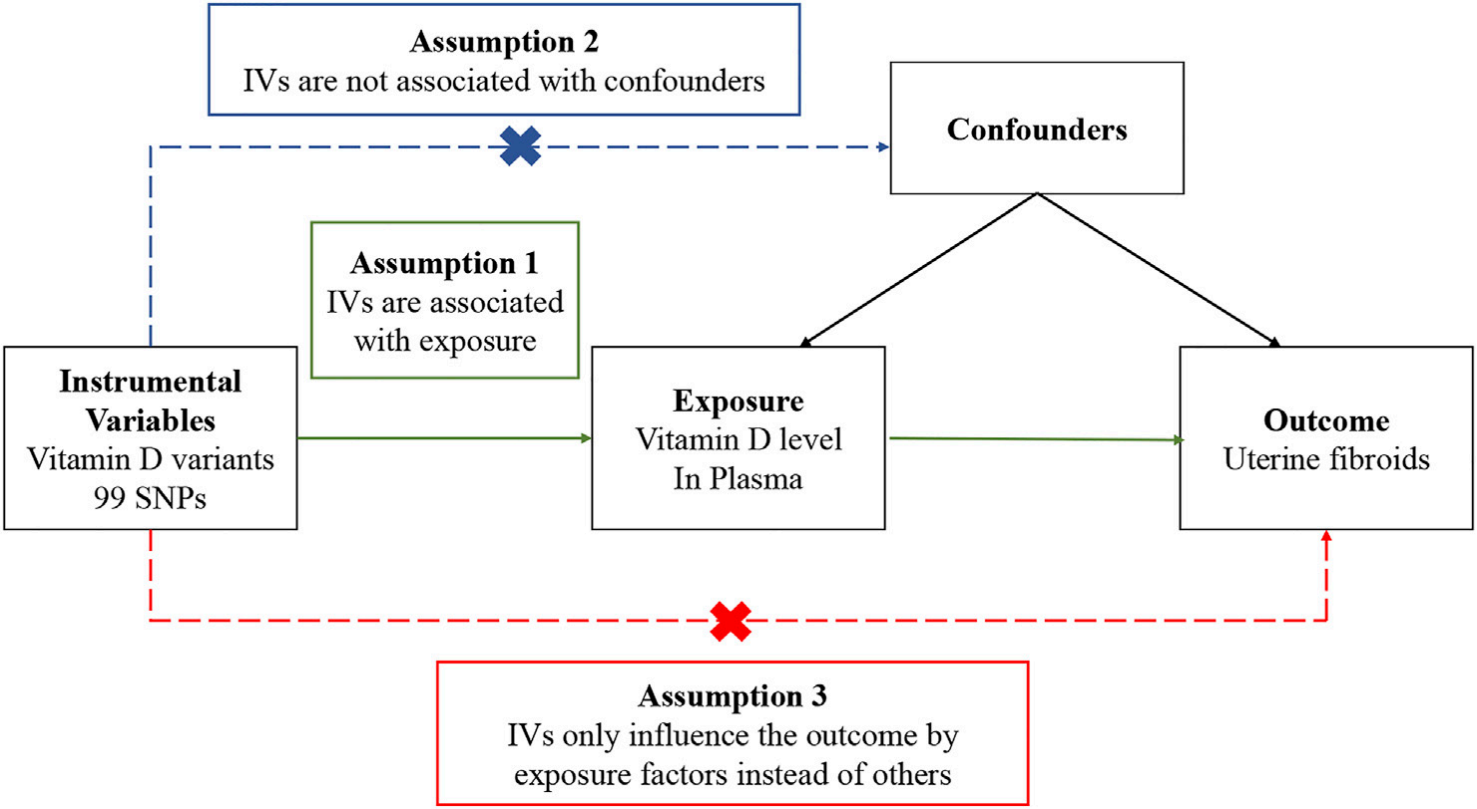
Overview of the design and three key assumptions of the Mendelian randomization study. IVs, instrument variables; SNPs, single-nucleotide polymorphisms.

In a Mendelian randomization study, to obtain reliable results, genetic variables as instrumental variables must satisfy three assumptions (Figure 1): 1) Genetic variation is associated with exposure factors; 2) Genetic variation is not associated with confounders; 3) Genetic variation only influences the outcome by exposure factors. The dashed line in the figure indicates that the pathway is not allowed, and the solid line indicates the ideal pathway. The second and third assumptions are collectively referred to as independence from pleiotropy. Pleiotropy refers to genetic variation that affects outcomes through pathways independent of risk factors. Investigators need to use sensitivity analysis to make judgments (Emdin et al., 2017).

### Genetic association dataset for Vitamin D

SNPs associated with vitamin D were selected from a genome-wide association study (GWAS) summary dataset, including 441,291 samples from European populations (Hemani et al., 2018), and the data were derived from research data including the United Kingdom biobank, FinnGen Biobank and other consortia, which were manually collected and organized into a summary dataset for use in MR studies. Independent SNPs that were related to vitamin D at the genome-wide significance level (*p* < 5 × 10^−8^) and low linkage disequilibrium (LD) level (r^2^ < 0.01) were selected. In order to avoid the possible bias caused by weak instrumental variables, we use the F-statistic to judge the strength of the instrumental variable (Burgess et al., 2011). According to experience, the F-statistic should be at least 10 (Staiger and Stock, 1997).

### Genetic instrumental vasriables for uterine fibroids

In this MR study, the occurrence of UFs was our outcome. Data for the outcomes were derived from the dataset provided to GWAS by the United Kingdom Biobank (Mitchell et al., 2019), containing 462,933 samples from European populations, of which 7122 were reported as UFs without other cancers. In this study, we extracted the effect estimates and standard errors for each of the 99 vitamin D–related SNPs from the GWAS summary statistics of UFs.

### Statistical analysis

MR analysis of the association between vitamin D and the occurrence of UFs was performed using 99 SNPs associated with 25-hydroxyvitamin D levels as IVs. We primarily performed MR analyses using Inverse-variance Weights (IVW) with random effects to estimate odds ratios (OR) and 95% confidence intervals (CI) for the occurrence of UFs (Burgess and Bowden, 2015).

We then performed a sensitivity analysis to examine heterogeneity and pleiotropy among IVs. MR-Egger regression, weighted median, simple model, and weighted model methods were used to determine whether IVs affected UFs through their effect on vitamin D alone. The slope coefficients of the MR-Egger regressions provided estimates of causal effects, which were used to test for pleiotropic bias (Bowden et al., 2015). Simple medians provide consistent estimates of causal effects if at least 50% of the IVs are valid, but weighted medians provide consistent estimates if at least 50% of the weights come from valid IVs (Bowden et al., 2016). And the weighted mode requires that the largest subset of instruments identifying the same causal effect estimates is contributed by valid IVs so that the result is consistent (Hartwig et al., 2017). We applied MR- Pleiotropy Residual Sum and Outlier (MR-PRESSO) analysis method to analyze the pleiotropy of IVs and correct the possible outliers. In addition, we use Q test on the IVW and MR-Egger to estimate the heterogeneity of the IVs. We also used a leave-one-out sensitivity test to test whether the MR outcome was sensitive to its related IV. MR and sensitivity analyses were performed in R (version 4.2.0) using the Two-Sample MR package (version 0.5.6) and the MRPRESSO package (version 1.0).

## Results

### SNPs used as instrumental variables

Independent SNPs that were related to 25-hydroxyvitamin D serum levels at the genome-wide significance level (*p* < 5 × 10^−8^) and low linkage disequilibrium (LD) level (r^2^ < 0.01) were selected from the GWAS dataset. Then SNPs with F > 10 were screened from these SNPs and the genes they are in were queried in Pubmed (https://www.ncbi.nlm.nih.gov/), and the genes of SNPs that did not belong to a specific gene were defined as NULL. The remaining 99 SNPs were included to establish the genetic IVs for vitamin D (Table 1).

**TABLE 1:** Vitamin D SNPs used to construct the instrument variable.

Chr	Position	SNP	Effect Allele	Other Allele	EAF	Beta	SE	Gene	*p* Value	F Statistics
1	41750648	rs12035012	A	C	0.222	0.014	0.002	HIVEP3	1.00E-200	882.53
1	62835936	rs12145455	C	T	0.095	0.019	0.003	ATG4C	1.00E-200	296.861
1	24609753	rs12403824	G	C	0.308	0.011	0.002	NULL	1.00E-200	1023.579
1	2315680	rs2843128	G	A	0.516	0.010	0.002	MORN1	1.00E-200	1319.309
1	151502427	rs34834099	T	C	0.066	0.026	0.004	CGN	1.00E-200	204.06
1	34684617	rs41266415	T	A	0.215	0.013	0.002	C1orf94	1.00E-200	790.3233
1	230293530	rs4631704	T	C	0.608	0.011	0.002	GALNT2	1.00E-200	1295.836
1	150522242	rs4971020	C	T	0.647	0.011	0.002	ADAMTSL4	1.00E-200	1162.396
1	46027355	rs512083	C	T	0.461	0.010	0.002	MAST2	1.00E-200	1309.729
1	62898984	rs60500353	T	C	0.156	0.015	0.003	LOC105378768	1.00E-200	541.4045
1	179423953	rs61826000	C	G	0.338	0.011	0.002	AXDND1	1.00E-200	1136.723
1	109807283	rs6657811	T	A	0.131	0.015	0.003	CELSR2	1.00E-200	425.2116
1	151037707	rs77152346	C	T	0.179	0.013	0.003	BNIPL	1.00E-200	583.828
2	21271707	rs34722314	A	T	0.136	0.017	0.003	NULL	1.00E-200	480.9317
2	27598097	rs4665972	C	T	0.606	0.017	0.002	ZNF512	1.00E-200	2042.473
3	52321788	rs13065677	T	C	0.051	0.023	0.004	DNAH1	1.00E-200	112.1106
3	153561145	rs1542926	C	T	0.117	0.016	0.003	NULL	1.00E-200	359.3695
3	173504091	rs16830473	C	T	0.091	0.018	0.003	NLGN1	1.00E-200	251.7777
3	85002871	rs1694929	T	C	0.430	0.010	0.002	CADM2	1.00E-200	1256.449
3	124687460	rs1909585	T	C	0.346	0.011	0.002	KALRN	1.00E-200	1109.83
3	47352998	rs76183418	C	T	0.179	0.013	0.003	KLHL18	1.00E-200	571.6435
3	125118082	rs7640441	A	C	0.241	0.013	0.002	SLC12A8	1.00E-200	922.0544
3	141654685	rs9861009	C	T	0.722	0.012	0.002	NULL	1.00E-200	1047.055
4	72501807	rs112001313	T	C	0.060	0.040	0.004	ADAMTS3	1.00E-200	260.385
4	71694520	rs11724493	C	T	0.056	0.022	0.004	GRSF1	1.00E-200	128.7095
4	69475763	rs12500806	T	C	0.613	0.012	0.002	NULL	1.00E-200	1064.943
4	100510550	rs145662623	A	G	0.061	0.020	0.004	EMCN	1.00E-200	144.4778
4	72179821	rs146216314	A	G	0.098	0.020	0.003	SLC4A4	1.00E-200	316.7743
4	70054650	rs28633736	C	T	0.149	0.016	0.003	HTN1	1.00E-200	531.4226
4	74458987	rs34169741	T	C	0.383	0.013	0.002	RASSF6	1.00E-200	1609.023
4	15892159	rs4364259	A	G	0.205	0.016	0.002	NULL	1.00E-200	879.1162
4	73681946	rs4694548	G	A	0.275	0.015	0.002	NULL	1.00E-200	1261.403
4	71489270	rs5020231	C	T	0.779	0.013	0.002	SLC4A4	1.00E-200	777.8031
4	72745430	rs62318873	T	C	0.052	0.050	0.005	NULL	1.00E-200	240.7028
4	72585683	rs71601778	C	T	0.074	0.082	0.004	NULL	1.00E-200	747.2279
4	73266887	rs72607843	T	C	0.329	0.012	0.002	ADAMTS3	1.00E-200	1214.048
4	87982876	rs7660883	G	C	0.378	0.012	0.002	SPP1	1.00E-200	1355.003
4	72396727	rs7683903	A	G	0.154	0.014	0.003	ADAMTS3	1.00E-200	506.9613
4	72599352	rs843004	G	A	0.076	0.039	0.004	NULL	1.00E-200	404.4502
5	87940026	rs10070734	C	T	0.704	0.012	0.002	LINC00461	1.00E-200	1097.487
5	118613707	rs13187496	G	T	0.345	0.011	0.002	LOC102467225	1.00E-200	1188.506
5	148007013	rs2068190	A	G	0.437	0.010	0.002	HTR4	1.00E-200	1313.18
6	80014585	rs13197862	A	G	0.130	0.015	0.003	TTK	1.00E-200	397.857
6	22755139	rs4466239	G	A	0.624	0.011	0.002	NULL	1.00E-200	1261.93
6	121854778	rs942380	G	A	0.595	0.011	0.002	NULL	1.00E-200	1407.937
7	106799997	rs257376	A	G	0.542	0.010	0.002	PRKAR2B	1.00E-200	1298.439
7	100798274	rs6970645	G	C	0.752	0.011	0.002	AP1S1	1.00E-200	835.7355
8	106470630	rs10101205	C	T	0.840	0.014	0.003	OXR1	1.00E-200	515.8738
8	143587121	rs12546526	C	T	0.859	0.015	0.003	EEF1D	1.00E-200	437.9549
8	30835535	rs2042073	G	A	0.604	0.010	0.002	TEX15	1.00E-200	1220.945
8	9168897	rs62493791	G	T	0.242	0.012	0.002	NULL	1.00E-200	841.1148
8	59370159	rs6985620	C	T	0.665	0.011	0.002	NULL	1.00E-200	1119.15
9	112239077	rs12554549	T	C	0.065	0.021	0.004	PTBP3	1.00E-200	159.146
9	107645674	rs62568181	C	T	0.104	0.016	0.003	ABCA1	1.00E-200	295.9726
9	125605840	rs7027254	C	T	0.147	0.014	0.003	MAPKAP1	1.00E-200	470.4589
9	35766116	rs7862695	T	C	0.411	0.010	0.002	NULL	1.00E-200	1245.768
10	5530385	rs11253202	C	T	0.211	0.012	0.002	NULL	1.00E-200	708.6447
10	118394551	rs2286779	C	G	0.519	0.010	0.002	PNLIPRP2	1.00E-200	1316.9
10	81965655	rs7900214	A	G	0.279	0.012	0.002	NRG3	1.00E-200	989.5996
11	15182597	rs1109326	T	G	0.542	0.014	0.002	INSC	1.00E-200	1859.66
11	13799056	rs111308232	A	G	0.054	0.026	0.004	NULL	1.00E-200	135.6992
11	70988410	rs1118116	G	A	0.205	0.018	0.002	SHANK2	1.00E-200	1018.199
11	65581135	rs11227307	A	G	0.647	0.012	0.002	EHBP1L1	1.00E-200	1280.435
11	2176852	rs11602347	G	C	0.407	0.010	0.002	INS-IGF2	1.00E-200	1227.656
11	117008946	rs504068	C	T	0.820	0.014	0.003	SIK3	1.00E-200	605.7437
11	75437630	rs575976	G	A	0.184	0.013	0.003	GDPD5	1.00E-200	612.6741
11	76469378	rs60651758	A	G	0.101	0.017	0.003	C11orf30	1.00E-200	305.5616
11	71849741	rs652197	T	C	0.864	0.016	0.003	FOLR3	1.00E-200	454.6313
12	38692203	rs1813392	C	T	0.537	0.010	0.002	CPNE8	1.00E-200	1368.937
12	111522026	rs7314285	G	T	0.068	0.022	0.004	ATXN2	1.00E-200	181.7633
12	96089917	rs78117488	T	C	0.062	0.021	0.004	NTN4	1.00E-200	145.9469
12	33692179	rs7958788	C	T	0.377	0.010	0.002	NULL	1.00E-200	1187.48
12	57979949	rs8873	A	G	0.223	0.012	0.002	KIF5A	1.00E-200	756.2545
13	60676803	rs7981402	A	G	0.340	0.010	0.002	LINC00378	1.00E-200	1112.565
14	39356756	rs17108517	A	G	0.056	0.021	0.004	LINC00639	1.00E-200	127.1741
14	103987078	rs2071408	A	G	0.366	0.012	0.002	TDRD9	1.00E-200	1306.471
15	77316131	rs12913937	A	G	0.354	0.010	0.002	PEAK1	1.00E-200	1137.209
15	58671559	rs1601933	T	C	0.468	0.014	0.002	ADAM10	1.00E-200	1793.52
15	90734426	rs34560261	T	C	0.170	0.014	0.003	BLM	1.00E-200	542.6187
15	58571401	rs72739147	T	A	0.130	0.016	0.003	NULL	1.00E-200	428.8216
16	11901557	rs11075016	G	A	0.285	0.012	0.002	GSPT1	1.00E-200	1053.374
16	72698702	rs1429435	G	C	0.942	0.022	0.004	LINC01572	1.00E-200	127.5589
16	4500544	rs2304634	T	C	0.686	0.011	0.002	HMOX2|	1.00E-200	1032.636
16	30878366	rs2878304	T	C	0.726	0.011	0.002	BCL7C	1.00E-200	955.8311
16	84734147	rs56158152	T	G	0.355	0.011	0.002	USP10	1.00E-200	1112.916
16	89882826	rs72631431	T	C	0.313	0.011	0.002	TCF25	1.00E-200	1021.177
16	70687185	rs77194050	G	A	0.052	0.023	0.004	IL34	1.00E-200	115.9125
17	66394054	rs9889884	C	T	0.756	0.013	0.002	PRKCA	1.00E-200	928.8681
19	11185919	rs10423733	C	T	0.181	0.015	0.003	KANK2	1.00E-200	684.9655
19	54658102	rs11606	G	C	0.426	0.011	0.002	CNOT3	1.00E-200	1333.397
19	53700807	rs11673357	C	T	0.761	0.014	0.003	NULL	1.00E-200	797.2597
19	51518297	rs2075695	G	A	0.560	0.011	0.002	KLK10	1.00E-200	1401.33
19	58348570	rs35805032	T	C	0.155	0.014	0.003	A1BG	1.00E-200	501.5067
19	19325963	rs3761077	T	G	0.111	0.017	0.003	MAU2	1.00E-200	357.4002
19	48323130	rs62131912	T	G	0.101	0.020	0.003	NULL	1.00E-200	351.4042
20	52728499	rs2616280	A	G	0.076	0.019	0.004	NULL	1.00E-200	188.9118
20	52687181	rs6127083	C	G	0.152	0.015	0.003	BCAS1	1.00E-200	531.8351
22	41081164	rs133075	T	G	0.553	0.010	0.002	NULL	1.00E-200	1306.933
22	31533796	rs2072193	C	G	0.061	0.027	0.004	SFI1|	1.00E-200	184.4545

Chr, chromosome; SNP, single-nucleotide polymorphism; EAF, effect allele frequency; SE, standard error.

### Mendelian randomization test results and data visualization

**FIGURE 2:**
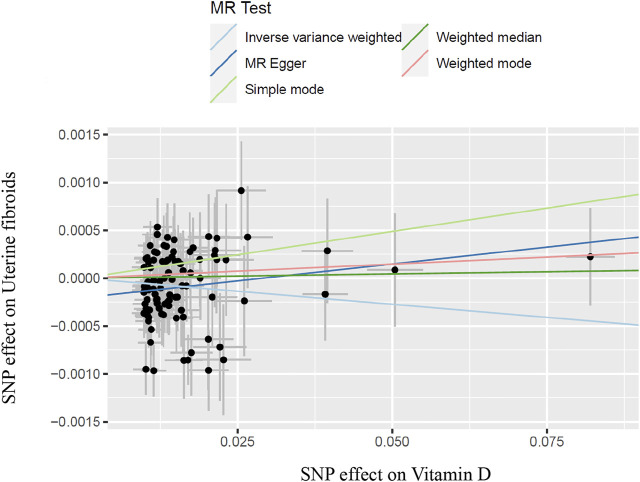
Scatter plot of SNPs with vitamin D and uterine fibroids and results of different test models; SNP, single-nucleotide polymorphism.

**FIGURE 3:**
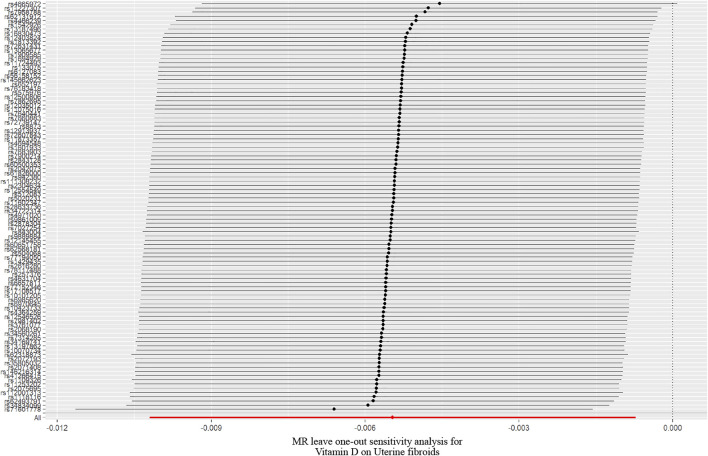
A leave one-out pleiotropy test was performed for each SNP to determine the robustness of the MR test; SNP, single-nucleotide polymorphism.

In the work, the IVW method was used to test for causal effects firstly. We found that a one-SD increase in vitamin D levels was associated with a decreased risk of UFs [odds ratio (OR): 0.995, 95% CI: 0.990-0.999, *p* = 0.024]. The result reveals the causal relationship between vitamin D levels and the risk of UFs in the European population. Then we adopted four different models to test and verify the causal relationship between serum vitamin D3 levels and UFs. All of the MR-Egger regression, weighted median, simple model, and weighted model results were opposite to IVW analysis (Table 2). Nevertheless, according to the judgment method of MR test results (Burgess and Thomson, 2015), our results are still able to draw the same conclusion. In addition, there has no bias value between our IVs in the scatter plot of correlation analysis (Figure 2) and the results of the leave-one-out sensitivity test (Figure 3), it illustrates that the causal relationship between vitamin D levels and the risk of UFs is highly reliable.

**TABLE 2:** Associations between genetically predicted vitamin D and risk of uterine fibroids.

Methods	OR (95% CI)	*p* Value
Inverse-variance Weighted	0.995 (0.999–0.990)	0.024
MR-Egger	1.007 (0.996–1.019)	0.216
Simple mode	1.010 (0.994–1.026)	0.225
Weighted median	1.001 (0.993–1.008)	0.814
Weighted mode	1.003 (0.993–1.013)	0.541

OR, odds ratio; CI, confidence interval.

### Heterogeneity and pleiotropy test results

To remove the possible bias of instrumental variables, heterogeneity test and horizontal pleiotropy test was conducted in the MR study. In sensitivity analysis, there have no heterogeneity was detected in the IVW method or the MR-egger method between IVs (*p* > 0.05) (Table 3). This means that our results are not confounded by other factors between populations grouped by IVs. But the intercept obtained by the MR-egger method was too far from 0, suggesting that there may be horizontal pleiotropy between the IVs (*p* < 0.05) (Table 4). Therefore, we performed multiple operations with MR-PRESSO and found no offset in IVs and no pleiotropy (*p* > 0.05). Furthermore, there have no outliers and horizontal pleiotropy were found after 2000 simulations using MR-PRESSO (Table 5). This indicates that the IVs used in this work impacted the risk of UFs only by affecting serum vitamin D3 levels.

**TABLE 3:** Heterogeneity testing of instrumental variables for vitamin D.

Method	Q	df	*p* Value
Inverse-variance weights	112.125	95	0.111
MR-egger	119.101	96	0.055

OR, odds ratio; CI, confidence interval.

**TABLE 4:** Pleiotropy testing of instrumental variables for vitamin D.

Method	Intercept	SE	*p* Value
MR-egger	2.05e-4	8.45e-5	0.017

SE, standard error.

**TABLE 5:** Pleiotropy testing of instrumental variables for vitamin D using MR-PRESSO.

	Exposure	MR Analysis	Casual Estimate	Sd	T-Stat	*p* Value
Main MR	VD	Raw	−5.27e−3	2.39e−3	−2.20	0.03
	VD	Outlier-corrected	NA	NA	NA	NA
RSSobs of Global Test in MR-PRESSO results:	123.669					
*p* value of Global Test in MR-PRESSO results:	0.063					

Sd, standard deviation; T-stat, T-statistics; VD, Vitamin D.

## Discussion

The Mendelian randomization study performed an analysis of the causal relationship between vitamin D and UFs based on a summary dataset from the United Kingdom Biobank including 462,933 individuals using multiple SNPs as instrumental variables. Our results revealed a causal relationship between serum vitamin D3 levels and the occurrence of UFs that the reduction of vitamin D levels will increase the risk of UFs.

Our findings are consistent with numerous previous studies. Early animal experiments found that vitamin D supplementation can significantly reduce the volume of UFs (Halder et al., 2010). A subsequent observational experiment showed that differences in serum vitamin D levels were significantly associated with the risk of developing UFs (Sabry et al., 2013), patients with lower vitamin D levels having a higher risk of developing UFs. These studies provide a potentially excellent therapeutic approach for the clinical treatment of UFs, which inspired researchers to further explore the phenomenon.

In the early 2000s, several studies revealed the mechanism of UFs—excessive secretion of compounds from extracellular matrix (ECM) such as collagen and fibers can cause UFs—and recent studies have also confirmed this (Sozen I and Arici, 2002; Rafique et al., 2017). The symptoms of ECM accumulation during the occurrence of UFs is similar to inflammation, which manifested as massive exudation of intracellular material and accumulation of ECM. Some researchers have proposed the possible involvement of inflammation in the development of UFs, and these processes are closely related to the function of vitamin D *in vivo* (Protic et al., 2016).

Vitamin D is a natural active substance, and its receptors are widely distributed *in vivo* and play different functions. Anti-inflammatory and anti-tumor effects are representative functions of Vitamin D (Van Den Bemd et al., 2000; Lips, 2006). Vitamin D generally exerts its biological function by regulating the level of growth factors through various signaling pathways. For example, Vitamin D is involved in the regulation of Wnt/β-catenin and TGF-β pathways (Ciebiera et al., 2017), which play important roles in the anti-inflammation and regulation of cell proliferation (Protic et al., 2016). Overexpression of TGF-β can lead to excessive secretion of ECM by stimulating the synthesis of collagen, proteoglycans, and other ECM compounds, which further induces the occurrence of UFs (Leppert et al., 2004; Ciebiera et al., 2017). Other studies also pointed out that increased vitamin D levels can suppress the cell proliferation and slow down the development of UFs by inhibiting Wnt/β-catenin and TGF-β pathways in the process of culturing UFs *in vitro* (Al-Hendy et al., 2016).

There are a series of factors participate in the regulation of cell proliferation and apoptosis, such as proliferating cell nuclear antigen (PCNA), cyclin-dependent kinase 1 (CDK1), M-phase promoting factor and catechol-O-methyltransferase (COMT), etc., Overexpression of these factors can promote the development of UFs by stimulating cell proliferation, which showing that vitamin D compounds can significantly inhibit the activation of enzymes that regulate factor expression and down-regulate the expression of them (Sharan et al., 2011). Furthermore, a study found that vitamin D can inhibit the expression of estrogen and progesterone receptors, and then suppress estrogen and progesterone perform endocrine functions (Al-Hendy et al., 2015). All of these evidences suggest that vitamin D play an important role in the development of UFs.

The researchers further conducted randomized clinical trials (RCT) to evaluate the effect of vitamin D supplementation on UFs. A clinical study, conducted on patients with small UFs and published in 2016, had reported a positive effect of vitamin D on fibroid volume reduction (Ciavattini et al., 2016). It indicated that vitamin D supplementation can significantly reduce the volume of UFs, but this is completely opposite to the results of another RCT (Arjeh et al., 2020). Interference of confounding factors and heterogeneity are also unavoidable in clinical trials. Thus, it is necessary to perform a MR study for further research. High-quality MR studies use Mendel’s law of random assignment and use SNPs as instrumental variables to minimize the influence of confounding factors and ensure that there is no heterogeneity in the study subjects, which would make the findings more convincing. Our MR study showed that low levels of vitamin D is associated with the increasing risk of UFs, which is consistent with the results of the former RCT (Ciavattini et al., 2016). We speculate that this is because the exposure simulated by the MR study is lifetime exposure, and the outcome is caused by chronically low serum vitamin D3 levels. On the other hand, vitamin D treatment within the RCT period is hardly produce enough therapeutic effects on normal-sized UFs compared to the life cycle, but it has better therapeutic effect on smaller-sized UFs.

The mechanism of action of vitamin D in the body is complex (Lips, 2006). This study found that some genetic variants can affect the risk of UFs through vitamin D3 serum levels. Genetic factors can affect vitamin D through multiple pathways (Hoffman et al., 2004), some non-vitamin D-related genes and their signaling pathways have been shown to play a role in promoting the development of uterine fibroids (Leppert et al., 2006), and this study can only explain some of the effects of vitamin D-related genetic variants on UFs. Therefore, vitamin D-related variants can only explain part of the risk of uterine fibroids, and other signaling pathways need to be analyzed to better understand other risk factors for uterine fibroids.

In the present work, we provide a scientific basis for further research on whether insufficient vitamin D is a causative risk factor for UFs, which may have important public health implications. Vitamin D is a natural component with high safety, relatively small side effects, high economic feasibility, and great research value. Further investigation of randomized clinical trials is needed to be constructed to actively explore the potential role of vitamin D or combination with other drugs on the treatment of UFs. This may help scientists develop a new generation of UFs treatment option (Al-Hendy and Badr, 2014).

One of the major strengths of study is the use of the MR study design, which can reduce the interference of confounders and determine the direction of causality. In addition, the study had large sample size, allowing us to examine more reliable causal association. Furthermore, we evaluate the consistency of the association through different methods to support the robustness of our results. However, several limitations in the study are also worth considering. First, our analysis is based on GWAS data from European populations, and the genetic variation among different races did not satisfy Mendel’s law of inheritance, so the obtained results may be difficult to extrapolate to the whole population. Second, our study did not investigate the therapeutic effect of vitamin D on UFs, although it established a causal relationship between vitamin D levels and UFs. Thirdly, The study examined serum vitamin D3 levels only through genetic pathways, and these genetic variants only play a role in specific contexts, given the complex biological role of the vitamin (Lips, 2006).

## Conclusion

In the MR study, we found that a one-SD decrease in serum vitamin D levels was associated with higher risk of UFs, consistent with previous studies describing a critical biological role for vitamin D in the development of UFs. Our study also implies the importance of adequate daily intake of vitamin D, which has positive effects on the prevention of UFs. However, due to the limited availability of evidence from clinical studies, further clinical studies are needed to explore the utility of vitamin D for the treatment of UFs.

## Data Availability

The original contributions presented in the study are included in the article/Supplementary Material, further inquiries can be directed to the corresponding author.
